# Serological Evidence of Widespread *Coxiella burnetii* Exposure Among Small Ruminants in Western Romania

**DOI:** 10.3390/vetsci13070698

**Published:** 2026-07-17

**Authors:** Timotei Pantea, Sebastian Alexandru Popa, Georgeta Stefan, Ionica Iancu, Vlad Iorgoni, Alexandru Gligor, Paula Nistor, Janos Degi, Luminita Costinar, Corina Pascu, Ionela Popa, Alexandru Udrea, David Purec, Cosmin Horatiu Maris, Viorel Herman

**Affiliations:** 1 Department of Infectious Diseases and Preventive Medicine, Faculty of Veterinary Medicine, University of Life Sciences “King Mihai I” from Timişoara, 300645 Timişoara, Romania; timoteipantea@yahoo.com (T.P.); ionica.iancu@usvt.ro (I.I.); vlad.iorgoni@usvt.ro (V.I.); alexandru.gligor@usvt.ro (A.G.); paula.nistor@usvt.ro (P.N.); janosdegi@usvt.ro (J.D.); luminita.costinar@usvt.ro (L.C.); corinapascu@usvt.ro (C.P.); udreaalexandru2000@yahoo.com (A.U.); david.purec.fmv@usvt.ro (D.P.); viorel.herman@fmvt.ro (V.H.); 2 Department of Animal Production and Veterinary Public Health, Faculty of Veterinary Medicine, University of Life Sciences “King Mihai I” from Timişoara, 300645 Timişoara, Romania; 3 Clinical Sciences Department, Faculty of Veterinary Medicine, University of Agronomic Sciences and Veterinary Medicine Bucharest, 050097 Bucharest, Romania; 4 Department of Semiology, Faculty of Veterinary Medicine, University of Life Sciences “King Mihai I” from Timișoara, 300645 Timisoara, Romania; ionela.popa@usvt.ro; 5 Department of Forestry, Faculty of Engineering and Applied Technologies, University of Life Sciences “King Mihai I” from Timișoara, 300645 Timișoara, Romania; cosmin.maris@usvt.ro; 6 Academy of Romanian Scientists, Str. Ilfov Nr. 3, Sector 5, 050044 Bucharest, Romania

**Keywords:** Q fever, zoonosis, small ruminants, ELISA, epidemiology, One Health, livestock surveillance

## Abstract

Q fever, caused by *Coxiella burnetii*, remains an important zoonotic disease with significant implications for animal health, livestock production, and public health. Epidemiological data regarding the circulation of *C. burnetii* in Romania remain limited, particularly in the western region of the country. This study aimed to assess the observed seropositivity and geographic distribution of anti-*C. burnetii* antibodies among sampled small ruminants from Western Romania. A cross-sectional sero-epidemiological survey was conducted using 546 serum samples collected from sheep (n = 451) and goats (n = 95) originating from five counties (Timiș, Arad, Bihor, Caraș-Severin, and Hunedoara). Sera were tested for IgG antibodies against *C. burnetii* using a commercial indirect enzyme-linked immunosorbent assay (ELISA). Overall, 87 animals were seropositive, corresponding to an observed seropositivity of 15.9% (87/546). Species-specific seropositivity was significantly higher in goats (25.3%) than in sheep (14.0%) (*p* = 0.006). Seropositive animals were detected in all surveyed counties, with prevalence ranging from 5.1% in Arad County to 21.3% in Timiș County. Significant geographic variation in seroprevalence was also observed among the investigated counties (*p* = 0.040). These findings indicate widespread serological evidence of exposure to *Coxiella burnetii* among the sampled small-ruminant population in Western Romania and reveal heterogeneous exposure patterns associated with both host species and geographic location. Continued surveillance integrating serological and molecular approaches is warranted to better characterize the epidemiology and transmission dynamics of *C. burnetii* within a One Health framework.

## Simple Summary

Q fever is a zoonotic disease caused by *Coxiella burnetii* that affects both animal and human health. Small ruminants, particularly sheep and goats, are recognized as important reservoirs of the pathogen and may contribute to environmental contamination and disease transmission. Information regarding the occurrence and distribution of *C. burnetii* in Romanian small-ruminant populations remains limited, especially in the western region of the country. In the present study, serum samples collected from sheep and goats in five counties of Western Romania were examined for antibodies against *C. burnetii* using a commercial ELISA assay. Evidence of previous exposure to the pathogen was identified in a considerable proportion of the tested animals, with significant differences observed between species and geographical areas. Seropositive animals were detected in all investigated counties, indicating geographically distributed serological evidence of previous exposure to *C. burnetii* within the sampled population. These findings provide updated epidemiological information and support the need for continued surveillance, strengthened biosecurity measures, and integrated strategies aimed at reducing the impact of Q fever on animal production and public health.

## 1. Introduction

Q fever is a globally distributed zoonotic disease caused by *Coxiella burnetii* (*C. burnetii*), an obligate intracellular bacterium capable of persisting in the environment for extended periods due to its highly resistant small-cell variant [1]. Domestic ruminants, particularly sheep, goats, and cattle, represent the primary reservoirs for human infection and play a central role in pathogen maintenance and dissemination [2]. Shedding occurs predominantly during parturition or abortion through placental tissues and birth fluids, but the organism may also be excreted in milk, feces, and urine. Transmission to humans occurs mainly through inhalation of contaminated aerosols, making areas characterized by high livestock density, intensive reproductive activity, or inadequate biosecurity particularly vulnerable to outbreaks [3,4,5].

Over the past two decades, numerous serological and molecular surveys across Europe have confirmed the endemic nature of *C. burnetii*, although prevalence varies widely across countries and production systems [5,6,7,8,9]. Studies from Western and Central Europe, including the Netherlands, Germany, France, and Hungary, report considerable spatial heterogeneity, with goats often exhibiting higher seroprevalence than sheep and cattle [4,5,6,7,8,9,10]. The large-scale human outbreak in the Netherlands (2007–2010) underscored the significant public health implications of unrecognized livestock circulation and highlighted the need for robust, region-specific monitoring programs [10,11].

In Romania, published data on Q fever epidemiology remain scarce and fragmented. A previous serological investigation documented exposure to *C. burnetii* in Romanian sheep and goats; however, available evidence remains limited and geographically restricted [12,13]. Given the country’s diverse livestock systems, which include extensive grazing, transhumance practices, and mixed-species farms, the actual distribution and magnitude of *C. burnetii* circulation remain insufficiently characterized [13]. Western Romania is of particular interest because recent epidemiological data on *C. burnetii* exposure in small-ruminant populations from this region are limited. The epidemiology of *C. burnetii* is influenced by complex interactions among animal hosts, environmental contamination, and human exposure. Previous studies have demonstrated the importance of integrating veterinary, environmental, and public health surveillance to better understand the epidemiology and transmission of Q fever across different epidemiological settings [14,15,16].

Updated epidemiological information is therefore essential to better understand current exposure levels, identify high-risk areas, and guide targeted prevention and surveillance strategies. In this context, the present study aimed to determine the observed seropositivity and geographic distribution of antibodies against *C. burnetii* among sampled small ruminants from five counties in Western Romania. By integrating species-level and spatial analyses, this study provides a contemporary assessment of serological exposure and contributes valuable baseline data for veterinary and public health planning in a region where recent, systematic data are largely lacking.

## 2. Materials and Methods

### 2.1. Study Design

A cross-sectional serological study was conducted between May and November 2025 to determine the observed seropositivity and geographic distribution of antibodies against *C. burnetii* among sampled small ruminants in Western Romania. The study included 546 serum samples obtained from sheep and goats originating from five counties: Arad, Bihor, Caraș-Severin, Hunedoara, and Timiș. The investigated population consisted of 451 sheep and 95 goats. No vaccination against Q fever was reported in any of the flocks of origin.

### 2.2. Study Area

The study was conducted in Western Romania and included five administrative counties: Timiș, Arad, Bihor, Caraș-Severin, and Hunedoara. These regions encompass diverse agro-ecological environments, including lowland plains, hilly landscapes, and submontane areas, and support a wide range of livestock production systems, ranging from extensive grazing systems to semi-intensive and intensive farming operations [17,18].

The study area is characterized by a temperate-continental climate, with mean annual temperatures ranging from approximately 9 to 12 °C and annual precipitation varying between 550 and 900 mm, depending on altitude and local geographic conditions.

The investigated region sustains substantial sheep and goat populations raised under diverse production systems, including extensive, semi-intensive, and intensive farming. Extensive grazing, communal pastures, seasonal animal movements, mixed-species farming, and contact between neighbouring flocks have been described as general characteristics of livestock production in parts of Western Romania [17,18,19]. These characteristics have been reported in the literature as typical for the region; however, they were not evaluated in the present study and therefore are provided only as background information. Seasonal animal movements, communal grazing practices, mixed-species farming, and frequent contact between neighboring flocks may facilitate the maintenance and transmission of *Coxiella burnetii*. These environmental and management-related characteristics provide a representative epidemiological setting for assessing the circulation and geographic distribution of *C. burnetii* among small ruminants in Western Romania [19,20,21].

**Figure 1:**
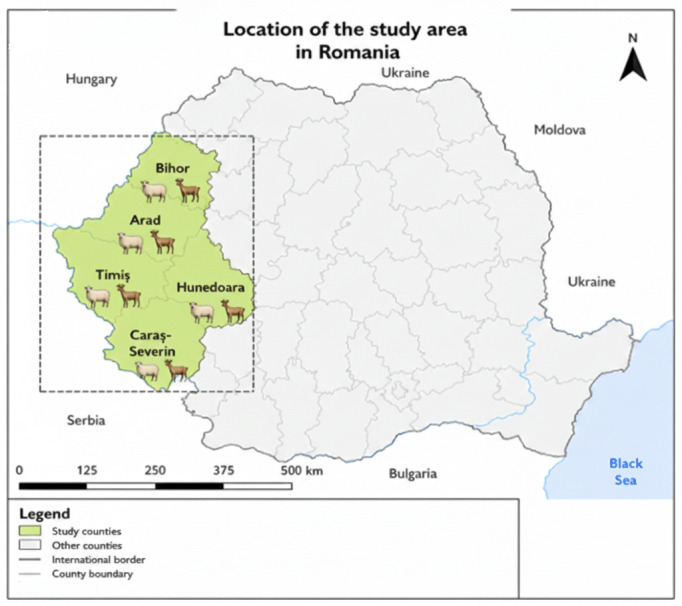
The geographic distribution of the study area.

A total of 546 serum samples were collected from sheep (n = 451) and goats (n = 95) originating from the five surveyed counties. The county-level distribution of sampled animals was as follows: Arad (n = 79; 61 sheep and 18 goats), Bihor (n = 84; 71 sheep and 13 goats), Caraș-Severin (n = 149; 124 sheep and 25 goats), Hunedoara (n = 126; 104 sheep and 22 goats), and Timiș (n = 108; 91 sheep and 17 goats). The geographic distribution of the study area and sampling locations is presented in Figure 1.

### 2.3. Sampling Strategy and Animal Selection

Sampling was performed using a convenience-based approach based on farm accessibility and owner consent, in accordance with epidemiological field investigation practices commonly applied in livestock production systems [22,23,24]. All sampled animals were clinically healthy at the time of blood collection. Animals originating from flocks with and without a recent history of reproductive disorders, particularly abortion, were eligible for inclusion to provide a broad assessment of serological exposure within the sampled population. Approximately 2–4% of the sampled animals originated from flocks with a recent history of abortion; however, none of the sampled animals showed clinical signs of reproductive disorders at the time of sampling. An additional inclusion criterion was that animals originated from flocks in which no vaccination against Q fever had been reported. Because a convenience-based sampling strategy was used, the seroprevalence estimates reported in this study refer only to the investigated study population and should not be interpreted as representative of the entire small-ruminant population of Western Romania.

Basic epidemiological information, including animal species and county of origin, was systematically available for all sampled animals. Information regarding reproductive history and husbandry practices was incomplete and was therefore not included in the statistical analyses.

No formal sample-size calculation was performed because this was an exploratory cross-sectional study based on convenience sampling. The number of animals included was determined by farm accessibility, owner consent, and the availability of animals during the study period.

### 2.4. Sample Collection and Processing

Blood samples were collected from sheep and goats by licensed field veterinarians through jugular venipuncture using sterile vacutainer tubes without anticoagulant. Following collection, samples were allowed to clot at room temperature and were subsequently centrifuged at 1500× *g* for 10 min to obtain serum.

Serum aliquots were transferred into sterile labeled cryovials and transported under refrigerated conditions (4–8 °C) to the diagnostic laboratory, where they were stored at −20 °C until serological testing [25,26,27].

All procedures related to sample collection, transportation, processing, and storage were performed according to standard veterinary laboratory practices in order to preserve sample integrity and ensure the reliability of subsequent serological analyses.

### 2.5. Serological Testing

Detection of IgG antibodies against *Coxiella burnetii* was performed using a commercial indirect enzyme-linked immunosorbent assay (ELISA), Monoscreen^®^ AbELISA *C. burnetii* (BIO K 298, Bio-X Diagnostics, Rochefort, Belgium), validated for use in sheep and goats.

The assay was performed according to the manufacturer’s instructions [28]. Serum samples were diluted 1:100 (10 µL serum and 990 µL dilution buffer) before analysis. One hundred microliters of diluted serum was added to each well and incubated for 60 min at 21 ± 3 °C, followed by three washing steps. Subsequently, 100 µL of diluted protein G-peroxidase conjugate was added and incubated for 60 min at 21 ± 3 °C. After a second washing step, 100 µL of TMB substrate was added for 10 min, and the reaction was stopped with 100 µL stop solution. Optical density was measured at 450 nm within 5 min after addition of the stop solution. Samples were analyzed in a single determination, and assay validity was verified using the manufacturer’s positive and negative controls. Results were interpreted according to the manufacturer’s S/P (%) cut-off values (negative <40%, doubtful 40–60%, positive >60%).

No doubtful results were obtained; therefore, all 546 serum samples were included in the final statistical analysis.

### 2.6. Data Management and Statistical Analysis

Serological results and associated epidemiological data were entered into a centralized database and verified for accuracy prior to statistical analysis. Overall, species-specific and county-specific seropositivity values were calculated as the observed proportion of ELISA-positive animals among the tested animals. The dependent variable was the serological status (positive/negative for *C. burnetii* antibodies), while the independent variables included animal species (sheep or goat) and county of origin.

Ninety-five percent confidence intervals (95% CI) were calculated for all prevalence estimates using the binomial method [29,30]. Differences in seroprevalence between sheep and goats were evaluated using the chi-square (χ^2^) test. Geographic variation in seroprevalence among the five surveyed counties was also assessed using the chi-square test, while Fisher’s exact test was applied when expected cell frequencies were below the accepted threshold [31].

Statistical significance was defined as *p* < 0.05. All statistical analyses and graphical representations were performed using GraphPad Prism version 10.0 (GraphPad Software, San Diego, CA, USA).

## 3. Results

A total of 546 serum samples collected from sheep and goats originating from five counties in Western Romania were analyzed for the presence of IgG antibodies against *Coxiella burnetii*. Serological testing identified 87 seropositive animals, corresponding to an overall observed seropositivity of 15.9% (87/546; 95% CI: 12.9–19.0). Seropositive animals were detected in both species and in all surveyed counties, demonstrating geographically distributed evidence of previous exposure within the sampled population.

### 3.1. Observed Seropositivity by Species

Species-specific analysis revealed differences in the seroprevalence of *C. burnetii* between sheep and goats. Among sheep, 63 of 451 animals were seropositive, corresponding to a seroprevalence of 14.0% (95% CI: 10.8–17.2). Among goats, 24 of 95 animals were seropositive, corresponding to a seroprevalence of 25.3% (95% CI: 16.6–34.1). Statistical analysis demonstrated a significant association between animal species and serological status (χ^2^ = 7.47; df = 1; *p* = 0.006), indicating significant variation in exposure to *C. burnetii* between the two species.

Detailed species-specific serological results, including the number of tested animals, seropositive animals, prevalence estimates, and corresponding 95% confidence intervals, are presented in Table 1.

### 3.2. Seropositivity by County

Seropositive animals were detected in all five surveyed counties. County-level seroprevalence ranged from 5.1% in Arad County to 21.3% in Timiș County. Intermediate prevalence values were recorded in Bihor (14.3%), Hunedoara (16.7%), and Caraș-Severin (18.1%).

County-specific seroprevalence estimates, together with the corresponding numbers of tested and seropositive animals and their 95% confidence intervals, are presented in Table 2.

Species-specific serological results, including the number of tested animals, the number of seropositive animals, and the corresponding seroprevalence by county, are presented in Table 3.

Species-specific analysis by county demonstrated heterogeneous patterns of observed seropositivity. Among sheep, the highest observed proportions were recorded in Timiș (18.7%), Caraș-Severin (16.9%), and Hunedoara (14.4%). Among goats, the highest observed proportion was recorded in Timiș (35.3%), followed by Hunedoara (27.3%) and Caraș-Severin (24.0%). These county-specific estimates should be interpreted cautiously because of the relatively small and unequal numbers of goats sampled across counties.

### 3.3. Statistical Analysis of Species and Geographic Differences

Statistical analysis demonstrated a significant association between animal species and serological status, with a higher observed proportion of seropositive animals among goats than among sheep (χ^2^ = 7.47; df = 1; *p* = 0.006). Significant geographic variation was also identified among the five surveyed counties (χ^2^ = 10.04; df = 4; *p* = 0.040). However, these unadjusted comparisons should be interpreted cautiously because species composition and sample size differed among counties, and no farm-level or multivariable adjustment was performed.

## 4. Discussion

The present study provides updated sero-epidemiological evidence supporting the endemic circulation of *C. burnetii* among small ruminants in Western Romania. The overall observed seropositivity of 15.9% confirms widespread exposure to the pathogen within the investigated region and is consistent with reports from other European countries where Q fever remains endemic but exhibits considerable spatial variability [12,21]. The detection of seropositive animals in all surveyed counties further highlights the broad geographic distribution of *C. burnetii* and its persistence within regional livestock production systems.

The overall seroprevalence observed in the present study is comparable to values reported in neighboring European countries, including Serbia, Hungary, and Greece, where seroprevalence estimates in small ruminants generally range between 10% and 30%, depending on production systems, animal management practices, and diagnostic methodologies [9]. Furthermore, the prevalence detected in the present investigation is broadly consistent with previous evidence of *C. burnetii* exposure reported in Romanian small-ruminant populations, supporting the continued circulation of the pathogen within the country.

A notable finding of this study was the marked geographical heterogeneity in the observed seropositivity among the surveyed counties, ranging from 5.1% in Arad County to 21.3% in Timiș County. Similar regional variation has been reported in other European studies, where livestock density, grazing practices, animal movements, environmental conditions, production systems, and farm management have been associated with differences in *C. burnetii* circulation. However, these variables were not systematically evaluated in the present study and therefore cannot be considered explanatory factors for the observed county-level differences. In addition, the unequal distribution of animal species among counties and the absence of farm-level or multivariable adjustment may have influenced the unadjusted geographic comparisons. Consequently, further analytical studies incorporating standardized flock-level, management, and environmental data are required to identify the determinants of the observed regional differences.

The observed geographic differences should also be interpreted in the context of regional livestock management practices. Western Romania comprises a mixture of extensive, semi-intensive, and intensive production systems, with frequent communal grazing, seasonal animal movements, mixed-species farming, and contact between neighboring flocks. These management characteristics may facilitate the maintenance and dissemination of *C. burnetii* through increased opportunities for animal-to-animal transmission and environmental contamination. Nevertheless, because detailed flock-level information regarding production systems and management practices was not consistently available for all sampled animals, their specific contribution to the observed county-level differences could not be formally evaluated and should be investigated in future epidemiological studies [4,15].

Species-specific differences also contributed to the understanding of *C. burnetii* epidemiology in the study area. Goats showed a higher observed proportion of seropositive animals than sheep (25.3% vs. 14.0%). Although this finding is consistent with several European serological studies, it should be interpreted cautiously because substantially fewer goats than sheep were sampled, the distribution of the two species differed among counties, and no farm-level or multivariable adjustment was performed. Several human Q fever outbreaks have been associated with infected goat herds, most notably the epidemic that occurred in the Netherlands between 2007 and 2010. Therefore, the present results identify an unadjusted association between animal species and serological status but do not establish that host species independently determined exposure.

Several methodological considerations should be taken into account when interpreting these findings. First, the cross-sectional design provides information on exposure at a single point in time and does not allow the assessment of temporal trends or causal relationships. Second, although the overall sample size was substantial, the unequal distribution of samples among counties may have affected the precision of county-level seroprevalence estimates, particularly in counties with smaller sample sizes, resulting in wider confidence intervals and reduced statistical power for regional comparisons [32]. Also, a limitation of the present study is the lack of complete and standardized information regarding husbandry practices, flock management, and biosecurity measures. Consequently, the potential influence of these factors on *C. burnetii* seropositivity could not be evaluated and should be addressed in future epidemiological investigations. Furthermore, the convenience-based sampling strategy limits the generalizability of the reported seroprevalence estimates beyond the investigated study population. Finally, while serological testing is an appropriate tool for assessing population-level exposure to *C. burnetii*, it does not distinguish between previous and active infection or bacterial shedding. Therefore, future studies integrating molecular approaches, such as PCR, would be valuable to identify actively infected animals and to further characterize the epidemiology of *C. burnetii* in Romanian small-ruminant populations [33,34,35,36,37].

Despite these limitations, the study provides useful baseline information for a region where contemporary data on Q fever in small ruminants remain limited. The geographically distributed detection of antibodies supports continued serological surveillance. Future investigations should integrate representative sampling, farm-level epidemiological data, molecular testing, and environmental sampling to assess active infection, bacterial shedding, and potential transmission pathways more accurately. Effective collaboration between veterinary and public health authorities will be essential for improving Q fever surveillance and mitigating its impact on both animal and human health.

## 5. Conclusions

The present study provides updated animal-level serological evidence of previous exposure to *C. burnetii* among sampled sheep and goats from five counties in Western Romania. Observed seropositivity differed according to animal species and county of origin, with a higher unadjusted proportion among goats than among sheep. These findings should be interpreted in the context of convenience sampling, unequal species distribution, absence of farm-level adjustment, and lack of molecular testing. Further studies based on representative sampling, standardized epidemiological variables, and molecular methods are needed to assess active infection, bacterial shedding, and transmission dynamics.

## Figures and Tables

**Table 1:** Species-specific seropositivity of *Coxiella burnetii* antibodies in small ruminants from Western Romania.

Species	No. Tested	Positive	Negative	Seropositivity % (95% CI)
Sheep	451	63	388	14.0 (10.8–17.2)
Goats	95	24	71	25.3 (16.6–34.1)
Total	546	87	459	15.9 (12.9–19.0)

**Table 2:** County-specific seropositivity of *Coxiella burnetii* antibodies in small ruminants from Western Romania.

County	No. Tested	Positive	Negative	Seropositivity % (95% CI)
Arad	79	4	75	5.1 (0.2–9.9)
Bihor	84	12	72	14.3 (6.8–21.8)
Caraș-Severin	149	27	122	18.1 (11.9–24.3)
Hunedoara	126	21	105	16.7 (10.2–23.2)
Timiș	108	23	85	21.3 (13.6–29.0)
Total	546	87	459	15.9 (12.9–19.0)

**Table 3:** Species-specific distribution of seropositive animals according to county of origin.

County	Sheep Tested	Sheep Positive n (%)	Goat Tested	Goat Positive n (%)
Arad	61	1 (1.6)	18	3 (16.7)
Bihor	71	9 (12.7)	13	3 (23.1)
Caraș-Severin	124	21 (16.9)	25	6 (24.0)
Hunedoara	104	15 (14.4)	22	6 (27.3)
Timiș	91	17 (18.7)	17	6 (35.3)
Total	451	63 (14.0)	95	24 (25.3)

## Data Availability

The original contributions presented in this study are included in the article. Further inquiries can be directed to the corresponding authors.

## Abbreviations

The following abbreviations are used in this manuscript:

ELISA	Enzyme-Linked Immunosorbent Assay
IgG	Immunoglobulin G
OD	Optical Density
TMB	Tetramethylbenzidine
PCR	Polymerase Chain Reaction
CI	Confidence Interval

## References

[1] España P.P., Uranga A., Cillóniz C., Torres A. (2020). Q fever (*Coxiella burnetii*). Semin. Respir. Crit. Care. Med..

[2] Tan T., Heller J., Firestone S., Stevenson M., Wiethoelter A. (2023). A systematic review of global Q fever outbreaks. One Health.

[3] Arricau-Bouvery N., Souriau A., Lechopier P., Rodolakis A. (2003). Experimental *Coxiella burnetii* infection in pregnant goats: Excretion routes. Vet. Res..

[4] Toledo-Perona R., Contreras A., Gomis J., Quereda J.J., García-Galán A., Sánchez A., Gómez-Martín Á. (2024). Controlling *Coxiella burnetii* in Naturally Infected Sheep, Goats and Cows, and Public Health Implications: A Scoping Review. Front. Vet. Sci..

[5] Körner S., Makert G.R., Ulbert S., Pfeffer M., Mertens-Scholz K. (2021). The Prevalence of *Coxiella burnetii* in Hard Ticks in Europe and Their Role in Q Fever Transmission Revisited—A Systematic Review. Front. Vet. Sci..

[6] Clark N.J., Soares Magalhães R.J. (2018). Airborne geographical dispersal of Q fever from livestock holdings to human communities: A systematic review and critical appraisal of evidence. BMC Infect. Dis..

[7] Van den Brom R., Moll L., van Schaik G., Vellema P. (2013). Demography of Q Fever Seroprevalence in Sheep and Goats in The Netherlands in 2008. Prev. Vet. Med..

[8] Schimmer B., Luttikholt S., Hautvast J.L.A., Graat E.A.M., Vellema P., van Duynhoven Y.T.H.P. (2011). Seroprevalence and Risk Factors of Q Fever in Goats on Commercial Dairy Goat Farms in The Netherlands, 2009–2010. BMC Vet. Res..

[9] Gache K., Rousset E., Perrin J.B., de Cremoux R., Hosteing S., Jourdain E., Guatteo R., Nicollet P., Touratier A., Calavas D. (2017). Estimation of the Frequency of Q Fever in Sheep, Goat and Cattle Herds in France: Results of a 3-Year Study of the Seroprevalence of Q Fever and Excretion Level of *Coxiella burnetii* in Abortive Episodes. Epidemiol. Infect..

[10] Abeykoon A.M.H., Clark N.J., Soares Magalhaes R.J., Vincent G.A., Stevenson M.A., Firestone S.M., Wiethoelter A.K. (2021). *Coxiella burnetii* in the Environment: A Systematic Review and Critical Appraisal of Sampling Methods. Zoonoses Public Health.

[11] Schneeberger P.M., Wintenberger C., van der Hoek W., Stahl J.P. (2014). Q Fever in The Netherlands—2007–2010: What We Learned from the Largest Outbreak Ever. Med. Mal. Infect..

[12] Christodoulou M., Malli F., Tsaras K., Billinis C., Papagiannis D. (2023). A Narrative Review of Q Fever in Europe. Cureus.

[13] Bărăităreanu S., Özdemir Y.-A., Dan M. (2018). Serological Detection of Anti-*Coxiella burnetii* Antibodies in Romanian Small Ruminants. Rev. Rom. Med. Vet..

[14] Wardrop N.A., Thomas L.F., Cook E.A., de Glanville W.A., Atkinson P.M., Wamae C.N., Fèvre E.M. (2016). The sero-epidemiology of *Coxiella burnetii* in humans and cattle, western Kenya: Evidence from a cross-sectional study. PLoS Negl. Trop. Dis..

[15] Zendoia I.I., Barandika J.F., Hurtado A., López C.M., Alonso E., Beraza X., Ocabo B., García-Pérez A.L. (2021). Analysis of environmental dust in goat and sheep farms to assess *Coxiella burnetii* infection in a Q fever endemic area: Geographical distribution, relationship with human cases and genotypes. Zoonoses Public Health.

[16] Crăcea E., Constantinescu S., Tofan N., Căruntu F., Dogaru D. (1989). Q fever urban cases in Romania. Arch. Roum. Pathol. Exp. Microbiol..

[17] Feher A., Adamov T., Raicov M. (2016). Analysis of Agriculture in the West Region of Romania. Rev. Agric. Rural Dev..

[18] Popovici E.-A., Damian N., Grigorescu I., Persu M. (2022). Indicator-based analysis of organic farming in Romania. Regional spatial patterns. Int. J. Agric. Sustain..

[19] Larson P.S., Espira L., Grabow C., Wang C.A., Muloi D., Browne A.S., Deem S.L., Fèvre E.M., Foufopoulos J., Hardin R. (2019). The sero-epidemiology of *Coxiella burnetii* (Q fever) across livestock species and herding contexts in Laikipia County, Kenya. Zoonoses Public Health.

[20] Wambua L., Bett B., Abkallo H.M., Muturi M., Nthiwa D., Nyamota R., Kiprono E., Kirwa L., Gakuya F., Bartlow A.W. (2025). National serosurvey and risk mapping reveal widespread distribution of *Coxiella burnetii* in Kenya. Sci. Rep..

[21] Vourvidis D., Kyrma A., Linou M., Edouard S., Angelakis E. (2021). Sero-epidemiology Investigation of *Coxiella burnetii* in Domestic Ruminants throughout Most Greek Regions. Vet. Med. Sci..

[22] Tamarozzi F., Legnardi M., Fittipaldo A., Drigo M., Cassini R. (2020). Epidemiological Distribution of *Echinococcus granulosus* s.l. Infection in Human and Domestic Animal Hosts in European Mediterranean and Balkan Countries: A Systematic Review. PLoS Negl. Trop. Dis..

[23] Espinosa L., Gray A., Duffy G., Fanning S., McMahon B.J. (2018). A scoping review on the prevalence of Shiga-toxigenic *Escherichia coli* in wild animal species. Zoonoses Public Health.

[24] Reis A.C., Ramos B., Pereira A.C., Cunha M.V. (2021). Global trends of epidemiological research in livestock tuberculosis for the last four decades. Transbound. Emerg. Dis..

[25] Canali D., Fijita A., Sluzula M., Vieira L., Nakatani S., Colombini M., Silva C., Dias C., Lopes A. (2024). A-150 Extended Pre-Analytical Stability Validation for Coagulation Tests in Brazilian Clinical Laboratory. Clin. Chem..

[26] Nakanishi R., Sakayori T., Matsui D., Kono M., Maki M., Dunois C., Komiyama Y., Morisaki H. (2025). Effects of Storage Time and Temperature on Coagulation Factor and Natural Anticoagulant Activities in Healthy Individuals. Sci. Rep..

[27] Elliott P., Peakman T. (2008). The UK Biobank Sample Handling and Storage Protocol for the Collection, Processing and Archiving of Human Blood and Urine. Int. J. Epidemiol..

[28] Bio-X Diagnostics (2023). Monoscreen^®^ AbELISA C. burnetii (BIO K 298), Instructions for Use.

[29] Hay J.A., Routledge I., Takahashi S. (2024). Serodynamics: A Primer and Synthetic Review of Methods for Epidemiological Inference Using Serological Data. Epidemics.

[30] DiCiccio T.J., Ritzwoller D.M., Romano J.P., Shaikh A.M. (2022). Confidence Intervals for Seroprevalence. Stat. Sci..

[31] Kim H.Y. (2017). Statistical notes for clinical researchers: Chi-squared test and Fisher’s exact test. Restor. Dent. Endod..

[32] Fincato A., Lucchese L., Bellinati L., Mazzotta E., Ragolia S., Asa’Ad S., Salata C., Natale A. (2025). Q Fever: Who Is at Risk? A Serological Survey in the General Population and Occupationally Exposed Individuals in Northern Italy. Pathogens.

[33] De França D.A., Kmetiuk L.B., Rodrigues O.J.D., Panazzolo G.A.K., Morikawa V., Duré A.Í.d.L., Langoni H., Fávero G.M., Biondo A.W. (2024). *Coxiella burnetii* (Q Fever) Exposure in Wildlife Professionals. Front. Public Health.

[34] Lemtudo A.P., Mutai B.K., Mwamburi L., Waitumbi J.N. (2021). Seroprevalence of *Coxiella burnetii* in patients presenting with acute febrile illness at Marigat District Hospital, Baringo County, Kenya. Vet. Med. Sci..

[35] Fomda B.A., Qadri U., Nazir M., Khan A.H. (2025). Molecular Insights into Q Fever: PCR-Based Detection of *Coxiella burnetii* from Clinical Specimens—A Retrospective Study from a Tertiary Care Hospital of North India. Vector-Borne Zoonotic Dis..

[36] García-Pérez A.L., Zendoia I.I., Ferrer D., Barandika J.F., Ramos C., Vera R., Martí T., Pujol A., Cevidanes A., Hurtado A. (2025). Combination of Serology and PCR Analysis of Environmental Samples to Assess *Coxiella burnetii* Infection Status in Small Ruminant Farms. Appl. Environ. Microbiol..

[37] Dobos A., Fodor I., Kiss G., Gyuranecz M. (2021). Serological survey of *Coxiella burnetii* infections in dairy cattle, sheep, goats and zoo animals in Hungary—Short communication. Acta Vet. Hung..

